# Impact of Adenomyosis and Endometriosis on Chronic Pelvic Pain after Niche Repair

**DOI:** 10.3390/jcm12103484

**Published:** 2023-05-16

**Authors:** Marie Timmermans, Michelle Nisolle, Géraldine Brichant, Laurie Henry, Evy Gillet, Betty Kellner, Stavros Karampelas

**Affiliations:** 1 Department of Obstetrics and Gynecology, CHU of Liège—Citadelle Site, University of Liège, 4000 Liège, Belgium; 2 Department of Obstetrics and Gynecology, Centre Hospitalier Universitaire Brugmann, Université Libre de Bruxelles, 1020 Brussels, Belgium

**Keywords:** adenomyosis, endometriosis, isthmocele, chronic pelvic pain

## Abstract

Chronic pelvic pain (CPP) is one of the main isthmocele symptoms, together with abnormal uterine bleeding and secondary infertility. When patients undergo a laparoscopic niche repair surgery, it is important to determine if they present associated pathologies, such as adenomyosis and/or endometriosis, which are also a cause of CPP. A retrospective study was performed on 31 patients with CPP undergoing a laparoscopic niche repair. The pre-operative ultrasound was analyzed to determine the presence of adenomyosis. Endometriosis was histologically diagnosed. CPP outcome was evaluated at early (3–6 months) and late (12 months) post-operative follow ups. In our population of 31 women presenting CPP, only six of them (19.4%) did not have any associated pathology. In the group of 25 patients with associated pathology, 10 (40%) had no benefit from the reconstructive surgery in terms of CPP at early follow-up (3–6 months) and 8 (32%) in the post-operative period at 12 months. Patients with CPP who undergo niche repair should be carefully selected as CPP does not seem to be a good indication for uterine scar repair in patients with concomitant adenomyosis and endometriosis.

## 1. Introduction

Chronic pelvic pain (CPP) has already been described by Alcock in 1926 [1], and it is defined as an intermittent or constant pain in the pelvis or in the lower abdomen, lasting for at least 6 months. It is sufficient to induce disability with impact on quality of life and/or on work productivity, requiring medical and/or surgical treatment [2]. In the population of reproductive-age women (from 18 to 49 years old), almost 15% of them complain of CPP [3]. This is often due to multifactorial components. The main causes are gynecological, gastro-intestinal, or urological diseases, while in some cases, other disorders, such as musculoskeletal, psychological, or neurological ones, could be involved [4]. Gynecological conditions that could induce CPP are inter alia endometriosis, adenomyosis, and isthmocele.

Endometriosis is characterized by the presence of endometrial cells outside the uterine cavity and affects about 10% of women in reproductive age. This pathology could present three different aspects: peritoneal, ovarian, and deep infiltrating endometriosis. The main symptoms are dyspareunia, dysmenorrhea, dyschezia, infertility, and CPP [3,5,6].

Adenomyosis is defined as a benign invasion of the endometrium through the adjacent myometrium [7]. It is now diagnosed in younger patients of reproductive age due to the progress in imaging, with a prevalence from 5% to 70% and, concomitantly, with endometriosis in 70% of the cases [8]. Symptoms vary from one patient to another, and their quality of life is usually impacted. While one third of the patients are asymptomatic, 50% of women with this disease suffer from menorrhagias, 30% from dysmenorrheas, and 2% from metrorrhagias. Some women experience CPP and dyspareunia [8,9,10].

The rate of Cesarean section (C/S) has been rapidly increasing over the last 30 years. Worldwide, from 2010 to 2018, it was estimated at a rate of 21.2% and extrapolated to reach 28.5% in 2030 [11]. Concomitantly, patients are facing more adverse effects linked to this surgical act. One of the C/S complications is the isthmocele, niche, or (as it is also called) Cesarean scar defect (CSD), first described by Poidevin in 1961 [12]. It is defined as an indentation of at least 2 mm into the myometrium at the site of a previous C/S incision [13]. It can be observed during transvaginal ultrasonography (TVUS) or saline infusion sonohysterography (SIS) as a triangular hypoechogenic area. Its prevalence varies from 24% to 70% using TVUS and from 56% to 84% using SIS [14].

Different risk factors have been identified for the development of an isthmocele, such as an incision in the lower uterine segment, often related to a labor lasting over 5 h or a cervical dilatation of 5 cm or above [14,15]. The presence of adhesions between scar and abdominal wall and/or retroverted uterus are also risk factors to develop niche [14,16]. In the majority of the cases, CSD is asymptomatic and diagnosed incidentally during TVUS [17]. One third (30%) of the patients are symptomatic and present one or more of the following characteristics: abnormal uterine bleeding (AUB (29–82%)) [18], dysmenorrhea (40–50%), chronic pelvic pain (CPP (35%)), dyspareunia (18%), and dysuria and secondary infertility [14,19]. Loss of muscular fibers at the scar site generates continuous inefficient painful contractions. Moreover the blood retained inside the isthmocele’s pouch could cause a local inflammation [20]. Secondary infertility could be explained by the presence of intra-uterine fluid, mucus, or blood accumulation at the cervix, preventing sperm penetration or embryo implantation [21]. In 30% of laparoscopic niche surgeries, endometriosis is observed and treated simultaneously [22].

The aim of this study is to determine if the presence of associated endometriosis and/or adenomyosis may affect the outcome of the surgical repair of niches in terms of chronic pelvic pain improvement. This study demonstrates that in one third of the cases, patients are not relieved from their CPP symptoms if they concomitantly present endometriosis and adenomyosis.

## 2. Materials and Methods

This single-center retrospective study was performed between 2016 and 2021 in the Department of Obstetrics and Gynecology of the CHU—site Citadelle, University of Liège, Belgium. The study was approved by the Hospital’s Ethics Committee under the reference number B412201836328.

The cohort included 31 women of age ≥18 years-old, with at least one previous cesarean section, who underwent a laparoscopic repair of their symptomatic isthmocele with an initial residual myometrial thickness (RMT) less than 5 mm. Isthmocele with its triangular hypoechogenic area was detected by TVUS and/or SIS and/or magnetic resonance imaging. RMT was measured on TVUS within the sagittal plane, where the residual myometrium was the thinnest. All patients presented pre-operatively CPP, with or without AUB, and/or not otherwise explained secondary infertility. CPP was considered when constant or intermittent pain lasted for longer than 6 months. Women with pain as dysmenorrhea, dyspareunia, dyschezia, and/or dysuria that could not be explained by any other pathology other than CSD were included.

We excluded patients for whom preoperative TVUS was not available or not evaluable. The pre-operative transvaginal ultrasonography was reviewed by authors (SK and EG) according to the MUSA criteria [23,24] to determine the presence and localization of adenomyosis and to classify as diffuse, focal, or well circumscribed (adenomyoma). Patients were considered to be suffering from adenomyosis if at least one of the following criterias was observed: a globular uterus, an asymmetrical thickening of the myometrium wall, an irregular or interrupted junctional zone, the presence of cysts and/or hyperechoic islands within the myometrium, the existence of echogenic subendometrial lines and buds, a fan-shaped shadowing, or translesional vascularity. Endometriosis was diagnosed based on histology and scored according to the revised American Society of Reproductive Medicine (rASRM) classification by author (MN) [25].

Laparoscopic surgery of niche repair was previously described by Karampelas et al. in 2021 [17], and is briefly summarized here. After a correct identification of the niche, the vesico-uterine peritoneum is detached and the bladder is carefully separated from the uterus. CO_2_ laser or cold scissors are used to open the scar, and the fibrotic scar tissue is resected up to the level where healthy myometrium is reached. Closing of the defect is realized by separated sutures to allow a double-layer closure. If endometriosis was observed during the reparation, the lesions were removed using CO_2_ laser.

According to the clinical practices, all patients underwent a gynecological examination at 3 to 6 months post-operatively, and afterwards at their annual check-up. Recurrence of CPP was concluded if the patient did not have any improvement of pain after surgery or if it reappeared within a year.

Data were collected from medical records.

Statistical analysis was conducted in R software (version 3.6.2) [26]. Quantitative variables were summarized with classical descriptive statistics. Qualitative variables were assessed using counts and percentages of subjects. The chi-squared test or the exact Fisher’s test was used for qualitative variables. Statistical significance was achieved at 95% confidence (*p*-value significance <0.05).

## 3. Results

Between 2016 and 2021, we identified 31 patients who underwent surgery for isthmocele and fulfilled the inclusion criteria.

The main characteristics of the population are presented in Table 1. The median population was 33 years old, with a gravidity and parity of two, a previous history of two Caesarean sections, and a BMI of 25 kg/m^2^. The median delay between last C/S and the isthmocele reparation was 4 years. Beyond the CPP experienced by all of the studied patients, the other main pre-operative symptoms were AUB (54.8%) and/or secondary infertility (45.2%). Twelve patients (38.7%) were under pre-operative hormonal treatment. The median pre-operative RMT was 1.8 mm.

Associated pathologies and related pre and postoperative CPP outcomes at early (3–6 months) and at 1-year follow-up are summarized in Table 2.

Our study population can be divided into four main groups as follows. Group 1 is composed of 15 patients (48.4%) suffering only from adenomyosis. Group 2, consisting of endometriosis alone, is represented by only one woman (3.2%). Group 3 is constituted of nine patients (29.0%) presenting, concomitantly, endometriosis and adenomyosis. Group 4 gathers six women (19.4%) with no other visible pathology except isthmocele. The groups suffering from adenomyosis (Groups 1 and 3) were analyzed in depth. In both groups, diffuse adenomyosis was the main observed type (*n* = 9 (60.0%) and *n* = 9 (100.0%), respectively for Groups 1 and 3). Looking at the endometriosis rASRM scoring, all stages were quite equally present in Group 3, except for Stage II, which represented 44.4%. During surgery, endometriotic lesions were removed in all cases.

Approximately 80% of our population presented adenomyosis combined or not with endometriosis (*n* = 24, 77.4%), and only six patients (19.4%) suffered from CPP without any other explanation than isthmocele. Regarding adenomyosis, no lesions, diffuse or focal, were treated surgically. The majority of our patients (18/24, 75%) had diffuse lesions, while 25% presented focal adenomyosis.

Concerning the 24 patients suffering from adenomyosis combined or not with endometriosis, 15 of them (62.5%) had no pre-operative hormonal treatment. At late post-operative evaluation (at 12 months), 10 women did not receive any hormonal treatment and only 3 of them suffered from CPP. A total of 45% (14/31) of these patients had the desire for pregnancy, and 57% (8/14) had a successful pregnancy.

At post-operative evaluation (early or late), 14/17 (82%) patients were relieved from their symptoms of AUB after niche repair. Two patients who are still experiencing AUB suffered from adenomyosis, which could explain the persistency.

In the early post-operative period (at 3–6 months), 15 out of 25 patients (60%) with either adenomyosis, endometriosis, or both concomitant pathologies were relieved from chronic pelvic pain. The patients who did not present CPP improvement show predominantly diffuse adenomyosis with or without endometriosis. In the late post-operative period at 12 months, recurrence or persistence of CPP was higher in patients with concomitant adenomyosis and endometriosis. In Group 4, two patients showed pain recurrence, which can be explained by the development of abdominal wall deep infiltrating endometriosis for one patient, and the presence of adenomyosis at 12 months for the other.

In summary, in our population of 31 women presenting CPP, only 6 of them (19.4%) did not have any associated pathology. In the group of 25 patients with associated pathology, 10 (40%) did not benefit from the reparation surgery in terms of CPP at early follow-up (3–6 months) and 8 (32%) in the post-operative period at 12 months

## 4. Discussion

In this study population of 31 women having a symptomatic isthmocele who underwent laparoscopic surgery reparation, one third were not relieved from their pain symptoms, especially if they presented concomitant adenomyosis, associated or not with endometriosis.

To our best knowledge, this is the first study about the recurrence of CPP after niche repair in patients suffering from adenomyosis.

When patients with isthmocele present metrorrhagias, CPP, or secondary infertility, different surgical techniques can be performed by vaginal repair, laparoscopy, hysteroscopy, or a combination of the last two techniques [27]. In the present study, laparoscopy was used.

In our population, we considered the concomitant endometriosis and/or adenomyosis as these pathologies that are also causing CPP. It is important to note that only 6 patients out of 31 (19.3%) did not present any associated pathology.

In our study population, we can confirm that adenomyosis was present before isthmocele surgery as it was visible on pre-operative TVUS. Nevertheless, it is impossible to determine whether adenomyosis was present before isthmocele apparition or if adenomyosis associated with C/S is an increased risk for isthmocele. For this purpose, it could be interesting to develop a prospective study that would compare patients prior to having the first Cesarean section and to screen the possible presence of adenomyosis. However, Riggs [28], in 2014, studied the link between Cesarean sections and adenomyosis, and found a strong association (OR 2.08).

One third of our population experienced CPP recurrence at 6 or 12 months post-operatively. In the population suffering from adenomyosis alone or combined to endometriosis, there are up to 41% of non-treated CPP at early post-operative visits, while if the patient has no associated pathology, it was only 16%. Our results are non-significant, but it is probably because our population is too small. Nevertheless, there is a paucity of publications with reference to adenomyosis and endometriosis in the case of isthmocele.

In the literature, there are only six publications where adenomyosis has been histologically detected in the resected scar tissue. No analyses by TVUS were performed.

In 1995, Morris analyzed pathologic findings on Caesarean scar defects and described iatrogenic adenomyosis in 28% of 51 hysterectomy specimens.

Gubbini et al. in 2011 [29] studied prospectively the reproductive outcome in 41 patients who underwent hysteroscopic surgery for isthmocele reparation. Almost half of the patients (46.3%) suffered from pelvic pain. After around 3 months, all patients said that pelvic pain was relieved. Adenomyosis was found in 4.9% of the resected tissue. All patients achieved a pregnancy within 12 to 24 months. However, there is no link between pelvic pain and adenomyosis. A pre-operative diagnosis of adenomyosis has not been investigated before the histology report of the resected tissue.

The present study demonstrated the absence of improvement in 30% of the cases, and this proposition was already observed in a previous publication of Dosedla [30]. Indeed, this prospective study recruited 11 women over a period of 3 years and focused on the effects of laparoscopic reparation for a period of 6 months. Almost 30% (3/11) of patients still suffer from pelvic pain. Once again, there is no mention of adenomyosis, nor endometriosis.

Vervoort in 2018 [31], in a prospective cohort study, included 101 women who underwent laparoscopic isthmocele resection and were followed for their main complaints. Slight reduction of dysmenorrhea was reported by 7 patients out of 11. There was no mention of adenomyosis. Some of the patients presented endometriosis. Vervoort hypothesized that pain relief could be explained by a reduction of blood accumulation in the niche and the decrease in the related uterine contractions.

Donnez in 2017 [32] studied 38 women after laparoscopic repair of isthmocele. Out of this study group, 91% were relieved of their pre-operative symptoms (bleeding and/or pain) but no long-term follow-up was performed. In this publication, endometriosis is mentioned in terms of the pathology report by analyzing the scar resected tissue. At histology of the resected tissue, endometriosis was observed in 21.1%.

In a retrospective study, Shapira [33] analyzed 67 patients who underwent hysteroscopic resection of symptomatic CSD between 2011 and 2016 and focused on AUB. In their population, only two patients suffered from pelvic pain. Adenomyosis was observed in seven (11.8%) resected tissues, and 63.4% women observed post-surgically reduced AUB.

Karampelas, in 2021 [17], included 31 patients who underwent laparoscopic repair of isthmocele. The success rates of the surgery as improvement of abnormal uterine bleeding, chronic pelvic pain, and secondary infertility were 71.4% (10 of 14), 83.3% (10 of 12), and 83.3% (10 of 12), respectively. None of these patients reported recurrency of their symptoms.

In 2022, Krentel et al. [19] identified retrospectively nine cases of uterine niche reparation or surgery between 2019 and 2021 due to symptoms or ectopic niche pregnancy. Five of them show adenomyosis in the resected niche tissue. Three of them present also concomitant endometriosis. The aim of this study was not CPP recurrency, as some patients underwent hysterectomy. They found that some specific symptoms as metrorrhagia, brownish spotting, dysmenorrhea, dyspareunia, and dysuria were linked to the presence of adenomyosis within the resected uterine niche.

A recent retrospective study, conducted by Piriyev [34] in 2022, reported the surgical technique to repair isthmocele in the case of fertility desire and the surgical outcomes. It included 28 patients between 2014 and 2020. Adenomyosis was observed in half of the patients (14 cases) during the laparoscopy and histologically confirmed in 43%. They also suggested that adenomyosis uteri could favor the formation of isthmocele.

The team of Ewies [35] studied 52 cases of hysterectomies, and 30 of them had no Cesarean section and 22 had a scared uterus. More than half of the cases (13/22) with a scarred uterus presented adenomyosis in the C/S scar resected tissue in histopathology.

In the literature, adenomyosis or endometriosis were histologically described and not detected before the surgery.

About isthmocele histopatholgy analysis, it has been described that isthmocele resected tissue presents unusual infiltration of local CD138(+) plasma cells [36]. This unusual infiltration is also found in case of chronic endometritis. This pathology, which is an endometrial inflammation, is often found associated with endometriosis and adenomyosis. Frequently asymptomatic, chronic endometritis could also be responsible for pain [37,38]. In our study, we did not investigate the infiltration of local CD138 in the resected scar tissue, but it could be interesting to evaluate the correlation of the CPP recurrence to high levels of CD138 in the resected scar tissue.

The strength of this study is the fact that we pre-operatively analyzed, by TVUS, the presence of adenomyosis. This is the first time that this kind of study has been performed. However, TVUS was reviewed retrospectively, and we had only a small number of patients (31). It could be very interesting to initiate a prospective study with a pre-operative imaging checkup in order to detect adenomyosis and/or endometriosis.

If the main complaint of the patient presenting an isthmocele is CPP, it is very important to investigate for concomitant adenomyosis and/or endometriosis, as one third of this population is not relieved from CPP after niche repair.

## 5. Conclusions

Cesarean section has an important impact on adenomyosis development. In our case series, 77% of our patients treated for isthmocele presented concomitant adenomyosis. Moreover, 75% of them showed diffuse or anterior focal adenomyosis.

To our best knowledge, this is the first long-term study on the CPP outcomes for patients who have been treated for niche repair. Our results demonstrate that there is an important CPP recurrence rate (one third) in patients with adenomyosis and concomitant endometriosis after 1 year. Patients who undergo niche repair should be selected carefully.

## Figures and Tables

**Table 1:** Main characteristics of the study population (*n* = 31) in median values (min–max) and preoperative symptoms expressed in number (and %); AUB: abnormal uterine bleeding, BMI: Body Mass Index, C/S: Caesarean section, RMT: residual myometrial thickness.

Main Characteristics	
Age (years)	33 (23–44)
BMI (kg/m^2^)	25 (18–37)
Gravidity	2 (1–6)
Parity	2 (1–4)
Previous C/S	2 (1–4)
Delay between last C/S and isthmocele surgery (years)	4 (1–18)
AUB	17 (54.8)
Secondary infertility	14 (45.2)
Preoperative hormonal treatment	12 (38.7)
RMT (mm)	1.8 (0.6–4.7)

**Table 2:** Description of the four different groups in terms of pathology at the time of pre-operative check-up and number of CPP recurrence at 3–6 months and at 12 months post-isthmocele reparation surgery (*n* (%)): AD: adenomyosis, CPP: Chronic Pelvic Pain, FU: follow-up, m: months, rASRM: revised American Society of Reproductive Medicine. Statistical Fisher’s Exact Test was performed.

	CPP Recurrence		
	Preop CPP	3–6 m FU	12 m FU
**Group 1—Adenomyosis only**	15 (48.4)	6 (40.0)	3 (20.0)
Diffuse AD	9 (60.0)	3	1
Focal anterior AD	3 (20.0)	0	0
Focal posterior AD	1 (6.6)	1	0
Focal fundus AD	1 (6.6)	1	1
Anterior Adenomyoma	1 (6.6)	1	1
**Group 2—Endometriosis only**—rASRM Stage I	1(3.2)	0	0
**Group 3—Concomitant AD and endometriosis**	9 (29.0)	4 (44.0)	5 (55.0)
Diffuse AD	9 (100.0)	4	5
Endometriosis rASRM Stage I	2 (22.2)	1	2
Endometriosis rASRM Stage II	4 (44.4)	2	2
Endometriosis rASRM Stage III	1 (11.1)	1	1
Endometriosis rASRM Stage IV	2 (22.2)	0	0
**Group 4—Absence of adenomyosis and endometriosis**	6 (19.4)	1 (16.7)	2 (33.3)
**Total**	31 (100.0)	11 (35.5)	10 (32.3)
***p*-value**		0.38	0.29

## Data Availability

The data presented in this study are available on request from the corresponding author.

## References

[1] Alcock A. (1926). Chronic Pelvic Pain in Women. Br. Med. J..

[2] Howard F. (2003). Chronic Pelvic Pain. Obstet. Gynecol..

[3] Brichant G., Moïse A., Nisolle M. (2022). Endometriosis as an inflammatory disease?. Rev. Med. Liege.

[4] Steege J.F., Siedhoff M.T. (2014). Chronic Pelvic Pain. Obstet. Gynecol..

[5] Nisolle M., Donnez J. (2019). Reprint of: Peritoneal endometriosis, ovarian endometriosis, and adenomyotic nodules of the rectovaginal septum are three different entities. Fertil. Steril..

[6] Boucher A., Brichant G., Gridelet V., Nisolle M., Ravet S., Timmermans M., Henry L. (2022). Implantation Failure in Endometriosis Patients: Etiopathogenesis. J. Clin. Med..

[7] Bird C.C., McElin T.W., Manalo-Estrella P. (1972). The elusive adenomyosis of the uterus—Revisited. Am. J. Obstet. Gynecol..

[8] Szubert M., Koziróg E., Olszak O., Krygier-Kurz K., Kazmierczak J., Wilczynski J. (2021). Adenomyosis and Infertility—Review of Medical and Surgical Approaches. Int. J. Environ. Res. Public Health.

[9] Struble J., Reid S., Bedaiwy M.A. (2015). Adenomyosis: A Clinical Review of a Challenging Gynecologic Condition. J. Minim. Invasive Gynecol..

[10] Munro M.G., Critchley H.O.D., Fraser I.S., Haththotuwa R., Kriplani A., Bahamondes L., Füchtner C., Tonye R., Archer D., Abbott J. (2018). The two FIGO systems for normal and abnormal uterine bleeding symptoms and classification of causes of abnormal uterine bleeding in the reproductive years: 2018 revisions. Int. J. Gynecol. Obstet..

[11] Betran A.P., Ye J., Moller A.-B., Souza J.P., Zhang J. (2021). Trends and projections of caesarean section rates: Global and regional estimates. BMJ Glob. Health.

[12] Poidevin L.O.S. (1961). The value of hysterography in the prediction of cesarean section wound defects. Am. J. Obstet. Gynecol..

[13] Nguyen A.D., Nguyen H.T.T., Duong G.T.T., Phan T.T.H., Do D.T., Tran D.A., Nguyen T.K., Nguyen T.B., Ville Y. (2022). Improvement of symptoms after hysteroscopic isthmoplasty in women with abnormal uterine bleeding and expected pregnancy: A prospective study. J. Gynecol. Obstet. Hum. Reprod..

[14] Kulshrestha V., Agarwal N., Kachhawa G. (2020). Post-caesarean Niche (Isthmocele) in Uterine Scar: An Update. J. Obstet. Gynecol. India.

[15] Iannone P., Nencini G., Bonaccorsi G., Martinello R., Pontrelli G., Scioscia M., Nappi L., Greco P., Scutiero G. (2019). Isthmocele: From Risk Factors to Management. Rev. Bras. Ginecol. E Obs. RBGO Gynecol. Obstet..

[16] Vikhareva Osser O., Valentin L. (2010). Risk factors for incomplete healing of the uterine incision after caesarean section: Caesarean scar defects. BJOG Int. J. Obstet. Gynaecol..

[17] Karampelas S., Salem Wehbe G., de Landsheere L., Badr D.A., Tebache L., Nisolle M. (2021). Laparoscopic Isthmocele Repair: Efficacy and Benefits before and after Subsequent Cesarean Section. J. Clin. Med..

[18] Tulandi T., Cohen A. (2016). Emerging Manifestations of Cesarean Scar Defect in Reproductive-aged Women. J. Minim. Invasive Gynecol..

[19] Krentel H., Lauterbach L.-K., Mavrogiannis G., De Wilde R.L. (2022). Laparoscopic Fluorescence Guided Detection of Uterine Niche—The Next Step in Surgical Diagnosis and Treatment. J. Clin. Med..

[20] Vissers J., Hehenkamp W., Lambalk C.B., Huirne J.A. (2020). Post-Caesarean section niche-related impaired fertility: Hypothetical mechanisms. Hum. Reprod..

[21] Vervoort A.J.M.W., Uittenbogaard L.B., Hehenkamp W.J.K., Brolmann H.A.M., Mol B.W.J., Huirne J.A.F. (2015). Why do niches develop in Caesarean uterine scars? Hypotheses on the aetiology of niche development. Hum. Reprod..

[22] Donnez O. (2020). Cesarean scar defects: Management of an iatrogenic pathology whose prevalence has dramatically increased. Fertil. Steril..

[23] Munro M.G. (2020). Classification and Reporting Systems for Adenomyosis. J. Minim. Invasive Gynecol..

[24] Van den Bosch T., Van Schoubroeck D. (2018). Ultrasound diagnosis of endometriosis and adenomyosis: State of the art. Best Pract. Res. Clin. Obstet. Gynaecol..

[25] Guzick D.S., Silliman N.P., Adamson G.D., Buttram V.C., Canis M., Malinak L.R., Schenken R.S. (1997). Prediction of pregnancy in infertile women based on the American Society for Reproductive Medicine's revised classification of endometriosis. Fertil. Steril..

[26] R Core Team (2022). R: A Language and Environment for Statistical Computing.

[27] Kremer T.G., Ghiorzi I.B., Dibi R.P. (2019). Isthmocele: An overview of diagnosis and treatment. Rev. Assoc. Médica Bras..

[28] Riggs J.C., Lim E.K., Liang D., Bullwinkel R. (2014). Cesarean section as a risk factor for the development of adenomyosis uteri. J. Reprod. Med..

[29] Gubbini G., Centini G., Nascetti D., Marra E., Moncini I., Bruni L., Petraglia F., Florio P. (2011). Surgical Hysteroscopic Treatment of Cesarean-Induced Isthmocele in Restoring Fertility: Prospective Study. J. Minim. Invasive Gynecol..

[30] Dosedla E., Calda P. (2017). Outcomes of Laparoscopic Treatment in Women with Cesarean Scar Syndrome. Med. Sci. Monit. Int. Med. J. Exp. Clin. Res..

[31] Vervoort A., Vissers J., Hehenkamp W., Brölmann H., Huirne J. (2017). The effect of laparoscopic resection of large niches in the uterine caesarean scar on symptoms, ultrasound findings and quality of life: A prospective cohort study. BJOG Int. J. Obstet. Gynaecol..

[32] Donnez O., Donnez J., Orellana R., Dolmans M.-M. (2016). Gynecological and obstetrical outcomes after laparoscopic repair of a cesarean scar defect in a series of 38 women. Fertil. Steril..

[33] Shapira M., Mashiach R., Meller N., Watad H., Baron A., Bouaziz J., Cohen S.B. (2020). Clinical Success Rate of Extensive Hysteroscopic Cesarean Scar Defect Excision and Correlation to Histologic Findings. J. Minim. Invasive Gynecol..

[34] Piriyev E., Schiermeier S., Römer T. (2022). Laparoscopic Isthmocele (Niche) Correction as prevention in patients with fertility desire. Ginekol. Polska.

[35] Ewies A.A.A., Qadri S., Awasthi R., Zanetto U. (2022). Caesarean section operation is not associated with myometrial hypertrophy–A prospective cohort study. J. Obstet. Gynaecol..

[36] Higuchi A., Tsuji S., Nobuta Y., Nakamura A., Katsura D., Amano T., Kimura F., Tanimura S., Murakami T. (2022). Histopathological evaluation of cesarean scar defect in women with cesarean scar syndrome. Reprod. Med. Biol..

[37] Kitaya K., Yasuo T. (2023). Commonalities and Disparities between Endometriosis and Chronic Endometritis: Therapeutic Potential of Novel Antibiotic Treatment Strategy against Ectopic Endometrium. Int. J. Mol. Sci..

[38] Khan K.N., Fujishita A., Ogawa K., Koshiba A., Mori T., Itoh K., Nakashima M., Kitawaki J. (2022). Occurrence of chronic endometritis in different types of human adenomyosis. Reprod. Med. Biol..

